# A Cohort Study on Cardiovascular Disease Mortality in Breast Cancer Patients With Different Subtypes

**DOI:** 10.1155/tbj/8076118

**Published:** 2026-04-18

**Authors:** Zhipeng Wang, Lihong Deng, Peng Wang, Yacong Zhang, Zhongyang Shen

**Affiliations:** ^1^ Institute of Transplantation Medicine, Tianjin First Central Hospital, Nankai University, Tianjin, China, nankai.edu.cn; ^2^ Institute of Preventive Medicine, Tianjin Centers for Disease Control and Prevention, Tianjin, China; ^3^ Department of Epidemiology and Biostatistics, Key Laboratory of Molecular Cancer Epidemiology, Tianjin, National Clinical Research Center for Cancer, Tianjin Medical University Cancer Institute and Hospital, Tianjin Medical University, Tianjin, China, tijmu.edu.cn; ^4^ Department of Epidemiology & Biostatistics, School of Public Health, Key Laboratory of Prevention and Control of Major Diseases in the Population, Tianjin Medical University, Ministry of Education, Tianjin, China, meb.gov.tr; ^5^ Tianjin Key Laboratory for Organ Transplantation, Tianjin First Central Hospital, Tianjin, China, tj-fch.com; ^6^ Organ Transplantation Centre, Tianjin First Central Hospital, Tianjin, China, tj-fch.com; ^7^ Key Laboratory of Transplantation, Tianjin First Central Hospital, Chinese Academy of Medical Sciences, Tianjin, China, cacms.ac.cn

**Keywords:** breast cancer, cardio-oncology, cardiovascular mortality, molecular subtype

## Abstract

**Purpose:**

To investigate cardiovascular disease (CVD) mortality among patients with different breast cancer (BC) subtypes to assess its implications for long‐term survival.

**Patients and Methods:**

In total, 423,758 BC patients were included in this study utilizing data from the Surveillance, Epidemiology, and End Results 17 Registries Database (2010–2020). Competing risk curves were utilized to assess whether the cumulative CVD mortality surpassed the cumulative BC mortality. Multivariate competing risk models were used to explore potential factors associated with CVD mortality. Standardized mortality ratios (SMRs) were calculated to investigate CVD mortality in comparison to the general population.

**Results:**

In total, 5863 BC patients died from CVD during follow‐up time, accounting for 10.0% of all deaths (56,856). The number of deaths and the percentage of all deaths among patients died from CVD for luminal A, luminal B, HER‐2 enriched, and triple‐negative subtype were 4160 (12.2%), 615 (9.7%), 291 (7.7%), and 797 (6.2%), respectively. After 9 years, BC mortality remained over 10% higher than CVD mortality for luminal A and B subtypes, but the difference was under 10% for HER‐2‐enriched and triple‐negative subtypes. HER‐2‐enriched and triple‐negative BC patients aged 55–84 had higher CVD mortality compared to the general population. Triple‐negative BC was independently associated with increased CVD mortality compared with luminal A (HR 1.103 [95% CI: 1.016–1.199]).

**Conclusions:**

Patients with the triple‐negative subtype have a high risk of CVD mortality. This underscores the need for enhanced surveillance and targeted cardiovascular interventions for these patients to improve their long‐term health outcomes.

## 1. Introduction

In 2023, an estimated 297,000 new cases of breast cancer (BC) and 43,000 deaths from BC occurred in the United States (US) [1]. It is expected that more than 4 million females in the United States have been diagnosed with BC, and this figure is expected to increase [2]. While BC‐specific mortality has decreased due to advancements in cancer treatment, the number of deaths from other causes, such as cardiovascular disease (CVD), has been increasing [3–6]. BC survivors have an increased risk of CVD mortality [3] due to either the toxicity of cancer treatment [7, 8] or their shared risk factors [4, 9].

BC is a heterogeneous disease. At least four subtypes have been robustly established following gene expression, including luminal A, luminal B, human epidermal growth factor receptor 2 (HER‐2)‐enriched, and triple‐negative BC [10, 11]. Each BC subtype has a distinct treatment strategy [12] and differs significantly in terms of risk factors [13, 14]. This implies that the risk of CVD mortality may vary among molecular subtypes of BC.

Previous studies have focused on the risk of CVD mortality among overall BC survivors [3, 15, 16] or investigated the increased risk of CVD development after exposure to certain treatments, such as anthracycline [17], trastuzumab [18], and radiotherapy [7, 8]. In light of possible treatment‐related cardiotoxic effects, a recent study reported that patients with HER‐2‐positive BC did not have increased heart‐specific mortality compared to patients with HER‐2‐negative BC among BC patients who received either chemotherapy or radiotherapy [19]. However, data on the expression of HER‐2 were unavailable until 2010 [11]. Further investigation is required for studies with longer follow‐up times. According to a recent study, the risk of fatal heart disease increased over time for most cancer survivors, including those with BC [20]. However, it is currently unclear whether there is a difference in the timing at which the medium‐ to long‐term risk of CVD mortality exceeds the mortality risk associated with different subtypes of BC within a certain time window. Understanding these timing differences is crucial for personalized follow‐up, optimizing treatment strategies by balancing cancer and cardiovascular risks and ultimately improving the quality of life for BC survivors. Our focus was mainly on exploring differences in CVD mortality rates among various molecular subtypes of BC rather than on investigating the impact of receiving chemotherapy or radiotherapy on CVD mortality. We included only patients who received chemotherapy or radiotherapy to ensure a homogeneous study group, reduce variability, and focus on the impact of these treatments on CVD mortality. This approach allows us to clearly assess the variation in CVD mortality across different subtypes and provides a foundation for future research to further explore how different treatment methods might influence CVD mortality in these subtypes. Therefore, the main purpose of this study was to investigate the differences in CVD mortality risk among patients with different subtypes of BC who received either chemotherapy or radiotherapy and to investigate whether CVD mortality exceeded BC mortality for patients with different subtypes of BC during the follow‐up period.

## 2. Materials and Methods

### 2.1. Study Population

The Surveillance, Epidemiology, and End Results (SEER) program of the National Cancer Institute is a network of population‐based incident tumor registries that includes data from 17 regional cancer registries throughout the USA [21, 22]. The SEER program includes uniformly reported data from selected population‐based cancer registries in the United States on patient demographics, month and year of diagnosis, tumor features, treatment usage, and mortality for all incident cancers. The SEER 17 data released in April 2023 were used [23], which covered approximately 26.5% of the entire US population [19]. The online WONDER database of the Centers for Disease Control and Prevention (CDC), which is representative of the whole US population, was selected as the reference cohort. (Centers for Disease Control and Prevention, National Center for Health Statistics. National Vital Statistics System, Mortality 1999–2020 on CDC WONDER Online Database, released in 2021. Data are from the Multiple Cause of Death Files, 1999–2020, as compiled from data provided by the 57 vital statistics jurisdictions through the Vital Statistics Cooperative Program accessed at https://wonder.cdc.gov/mcd-icd10.html on Apr 2, 2024 5:30:08 AM.) [24]. These national mortality and population statistics at the county level are derived from US resident death certificates. To ascertain the cause of death, SEER data were combined with information from the National Center for Health Statistics. Survival time, as recorded in months, started at the date of cancer diagnosis and ended at the date of death, last known to be alive, or at the end of follow‐up, whichever came first. Since the data on the expression of HER‐2 were unavailable until 2010, participants identified after 2009 were eligible for this study. A total of 633,160 BC patients were initially identified. After excluding 4639 patients with an unknown stage, 6 patients aged under 18 years old, and 8196 patients with a follow‐up time of less than 1 month, 620,319 patients were identified. Overall, 423,758 (68.3%) patients who received either radiotherapy, chemotherapy, or both were eligible for this study (Supporting Figure 1).

### 2.2. Outcome and Covariate Assessment

According to a previous study [25], based on the SEER Causes of Death Register and International Classification of Diseases (10^th^ Revision, ICD10), CVD death included death from heart disease (ICD10: I00–I09, I11, I13, I20–I51), hypertension without heart disease (ICD10: I10, I12), cerebrovascular disease (ICD10: I60–I69), atherosclerosis (ICD10: I70), and other diseases of the circulatory system (I71–I78). Cancer‐related deaths were defined as deaths from BC. Other causes of death were defined as deaths that did not result from CVD or BC. Covariates such as age (categorized as age < 50, 50–64, 65–74, > 74 years), race (white, black, other (including Asian, Pacific Islander, American Indian, and Alaskan Native), unknown), SEER history at diagnosis (localized/in situ, regional, distant), type of reporting source (hospital inpatient/outpatient or clinic, other/unknown), year of diagnosis (2010–2014, 2015–2020), and molecular subtypes of BC (luminal A, luminal B, HER‐2‐enriched, triple‐negative) were considered potential factors of mortality. BC has been classified into four major subtypes: luminal A (ER[estrogen receptor]+ and/or PR[progesterone receptor]+, HER‐2−), luminal B (ER+ and/or PR+, HER‐2+), HER‐2‐enriched (ER−, PR−, HER‐2+), and triple‐negative (ER−, PR−, HER‐2−). This classification was based on the SEER‐derived BC subtype variable. Hormone receptor (HR) status was defined as positive (HR+) if either ER or PR was recorded as positive or borderline, aligning with contemporary guidelines that use lower positivity thresholds (e.g., ≥ 1%). HER‐2 status was determined using the SEER “Derived HER‐2 Recode,” which integrates HER‐2 results across the study period (2010–2020) through a validated algorithm. This algorithm prioritizes results from fluorescence in situ hybridization (FISH), chromogenic in situ hybridization (CISH), or other tests over immunohistochemistry (IHC) findings: cases with a positive result from any of these methods are classified as HER‐2 positive; among the remaining cases, those with a negative result are classified as HER‐2 negative. Cases with borderline HER‐2 status, along with those where HR or HER‐2 status was unknown, missing, or not done, were excluded from the subtype analysis. Details of the derived HER‐2 variable can be obtained from the SEER website (https://seer.cancer.gov/seerstat/databases/ssf/her2-derived.html).

### 2.3. Statistical Analysis

The baseline descriptive demographic characteristics of patients with different molecular subtypes of BC for categorical variables were compared using Pearson’s chi‐squared (*χ* ^2^) tests. Then, Fine–Gray tests were used to select potential predictors associated with CVD mortality in patients with different molecular subtypes of BC. Temporal trends in the proportions of CVD death, BC death, and other causes of death were utilized to demonstrate the preliminary correlations between these three causes of death in patients with different subtypes of BC. After that, cumulative incidence functions based on the Fine–Gray hypothesis were carried out to investigate when the cumulative CVD mortality rate began to outweigh the cumulative BC mortality rate since diagnosis. All samples with missing values were excluded. Multivariate competing risk models based on Fine–Gray tests were further performed to investigate the independent factors associated with CVD mortality in patients with different molecular subtypes of BC [26]. The multivariate competing risk model included the following covariates: demographic characteristics (age at diagnosis, categorized as < 50, 50–64, 65–74, and > 74 years; and race, categorized as White, Black, and Other [including Asian, Pacific Islander, American Indian, and Alaskan Native]); tumor characteristics (SEER historic stage at diagnosis [localized/in situ, regional, distant] and molecular subtype [luminal A, luminal B, HER‐2‐enriched, triple‐negative]); and healthcare process variables (year of diagnosis [2010–2014 vs. 2015–2020]; and type of reporting source [hospital inpatient/outpatient or clinic vs. other/unknown]). The luminal A subtype was used as the reference group for molecular subtype comparisons. To determine the associations between risk factors and CVD mortality, hazard ratios (HRs) and 95% confidence intervals (95% CIs) were estimated using multivariate competing risk models. Standardized mortality ratios (SMRs) were calculated to estimate CVD mortality risk in patients with different molecular subtypes of BC with the US female standard population from the CDC database as a reference [24]. All analyses were performed with R software (version 4.3.0). Sensitivity analyses were conducted including BC patients whose follow‐up time was longer than 3 months. All the statistical analyses were two‐sided, and *p* values less than 0.05 were considered to indicate statistical significance.

## 3. Results

### 3.1. CVD Mortality in BC Patients

In total, 5863 BC patients died from CVD, accounting for 10.0% of all deaths (56,856). The median follow‐up time for BC patients treated with radiotherapy or chemotherapy was 4.25 years (interquartile range [IQR] 2.00–7.08). Patients with luminal A BC were younger on average and diagnosed at an earlier stage than patients with other subtypes of BC. Patients with triple‐negative BC had a worse overall survival rate than patients with other subtypes of BC (Table 1). We observed that older age at diagnosis was associated with an increased percentage of patients who died from CVD among all four subtypes of BC patients (all *p* values < 0.001, Supporting Table 1).

**TABLE 1:** Cardiovascular mortality risk in different subtypes of breast cancer patients by baseline characteristics^a^.

Variable	Luminal A BC	Luminal B BC	HER‐2‐enriched BC	Triple‐negative BC
Total number	292,655	52,831	22,354	55,918
Age, mean (SD)	60.31 (11.94)	56.42 (12.73)	57.03 (12.48)	57.59 (13.17)
Age at diagnosis, years, *n* (%)				
< 50	58,611 (20.0)	16,122 (30.5)	6044 (27.0)	15,637 (28.0)
50–64	118,993 (40.7)	22,030 (41.7)	10,150 (45.4)	22,499 (40.2)
65–74	80,738 (27.6)	10,313 (19.5)	4138 (18.5)	11,942 (21.4)
74+	34,313 (11.7)	4366 (8.3)	2022 (9.0)	5840 (10.4)
Race, *n* (%)				
White	235,215 (80.4)	40,210 (76.1)	15,917 (71.2)	39,628 (70.9)
Black	26,872 (9.2)	6025 (11.4)	3119 (14.0)	11,361 (20.3)
Other (American Indian/AK Native, Asian/Pacific Islander)	28,976 (9.9)	6314 (12.0)	3146 (14.1)	4640 (8.3)
Unknown	1592 (0.5)	282 (0.5)	172 (0.8)	289 (0.5)
Type of Reporting Source, *n* (%)				
Hospital inpatient/outpatient or clinic	279,607 (95.5)	50,864 (96.3)	21,444 (95.9)	53,633 (95.9)
Other/Unknown	13,048 (4.5)	1967 (3.7)	910 (4.1)	2285 (4.1)
Year of diagnosis, *n* (%)				
2010–2014	123,538 (42.2)	21,802 (41.3)	9663 (43.2)	24,224 (43.3)
2015–2020	169,117 (57.8)	31,029 (58.7)	12,691 (56.8)	31,694 (56.7)
SEER historic stage, *n* (%)				
Localized/In situ	184,280 (63.0)	28,508 (54.0)	10,688 (47.8)	33,185 (59.3)
Regional	94,885 (32.4)	20,050 (38.0)	9186 (41.1)	18,919 (33.8)
Distant	13,490 (4.6)	4273 (8.1)	2480 (11.1)	3814 (6.8)
ICD‐10 site, *n* (%)				
C50.0 (Nipple)	806 (0.3)	193 (0.4)	102 (0.5)	98 (0.2)
C50.1 (Central portion of breast)	13,191 (4.5)	2558 (4.8)	1043 (4.7)	1639 (2.9)
C50.2 (Upper‐inner quadrant of breast)	38,110 (13.0)	5905 (11.2)	2324 (10.4)	7247 (13.0)
C50.3 (Lower‐inner quadrant of breast)	16,052 (5.5)	2861 (5.4)	1161 (5.2)	3264 (5.8)
C50.4 (Upper‐outer quadrant of breast)	103,144 (35.2)	17,407 (32.9)	7323 (32.8)	21,250 (38.0)
C50.5 (Lower‐outer quadrant of breast)	22,703 (7.8)	4363 (8.3)	1703 (7.6)	4006 (7.2)
C50.6 (Axillary tail of breast)	1533 (0.5)	291 (0.6)	101 (0.5)	407 (0.7)
C50.8 (Overlapping lesion of breast)	68,335 (23.4)	12,135 (23.0)	5012 (22.4)	12,246 (21.9)
C50.9 (Breast, unspecified)	28,781 (9.8)	7118 (13.5)	3585 (16.0)	5761 (10.3)
Cause of death, *n* (%)				
Alive	258,724 (88.4)	46,503 (88.0)	18,579 (83.1)	43,096 (77.1)
Breast cancer	18,105 (6.2)	3985 (7.5)	2633 (11.8)	9390 (16.8)
Cardiovascular disease	4160 (1.4)	615 (1.2)	291 (1.3)	797 (1.4)
Other	11,666 (4.0)	1728 (3.3)	851 (3.8)	2635 (4.7)

Abbreviations: BC, breast cancer; CVD, cardiovascular disease; ICD, International Classification of Diseases.

^a^ *p*‐values for differences in categorical variables between different subtypes of breast cancer patients are all < 0.001.

### 3.2. Temporal Trends in the Proportions of Cardiovascular and Cancer Mortality

The proportion of CVD‐related mortality in patients with luminal A BC increased from 8.8% in the first year after cancer diagnosis to 17.4% after 9 years, while the proportion of cancer‐related mortality decreased from 69.2% to 33.9%. The proportions of CVD mortality did not exceed the proportions of BC mortality, where the number of deaths from BC was > 10% greater than the number of deaths from CVD. A similar trend was observed in patients with luminal B BC. The proportions of CVD mortality in patients with triple‐negative BC increased from 5.2% in the first year after cancer diagnosis to 19.7% after 9 years, while the proportions of BC mortality decreased from 75.0% to 27.1%. The proportions of CVD mortality did not exceed the proportions of BC mortality, where the number of deaths from BC was < 10% greater than the number of deaths from CVD. A similar trend was observed in patients with HER‐2‐enriched BC (Figure 1, Supporting Figure 2).

FIGURE 1Temporal trends in the proportions of cardiovascular disease mortality and breast cancer mortality in different subtypes of breast cancer patients. Note: CVD, cardiovascular disease. The figure illustrates the temporal trends in the proportions of cardiovascular disease mortality and breast cancer mortality in four subtypes of breast cancer patients by using line graphs at different time points. After 9 years of breast cancer diagnosis, for patients with luminal A and luminal B subtypes, the proportion of patients with breast cancer mortality was still more than 10% higher than that of patients with cardiovascular disease, while the differences were smaller for patients with HER‐2‐enriched and triple‐negative subtypes.

**Figure:**
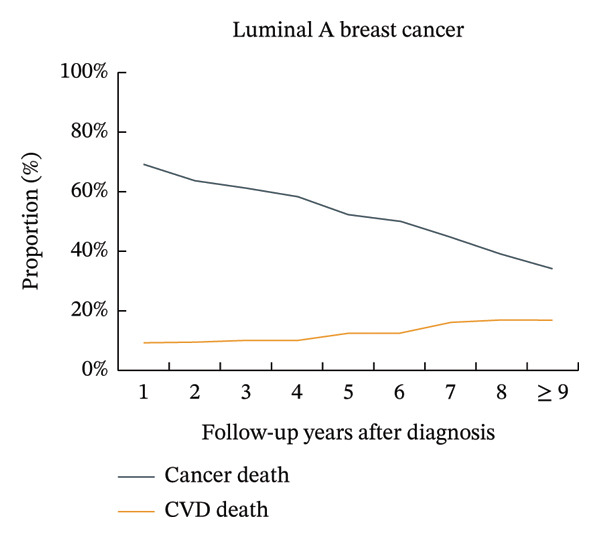
(a)

**Figure:**
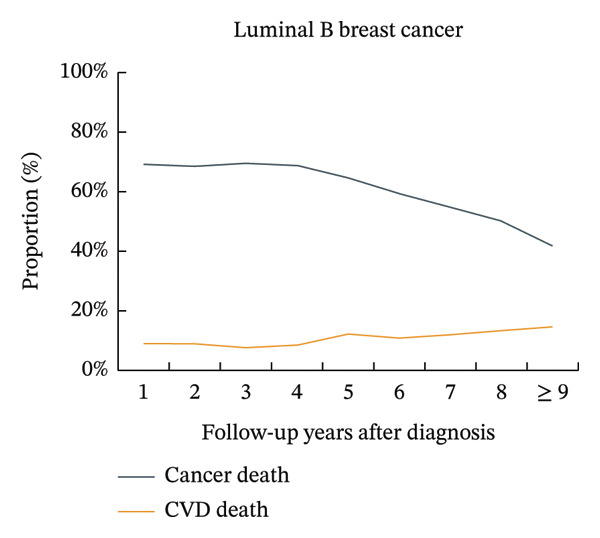
(b)

**Figure:**
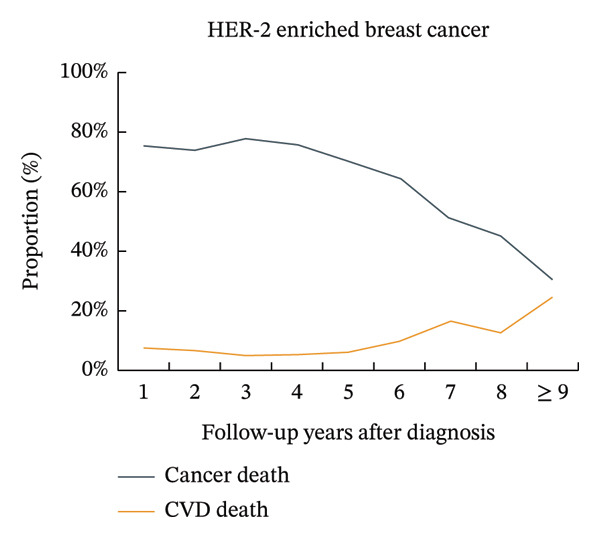
(c)

**Figure:**
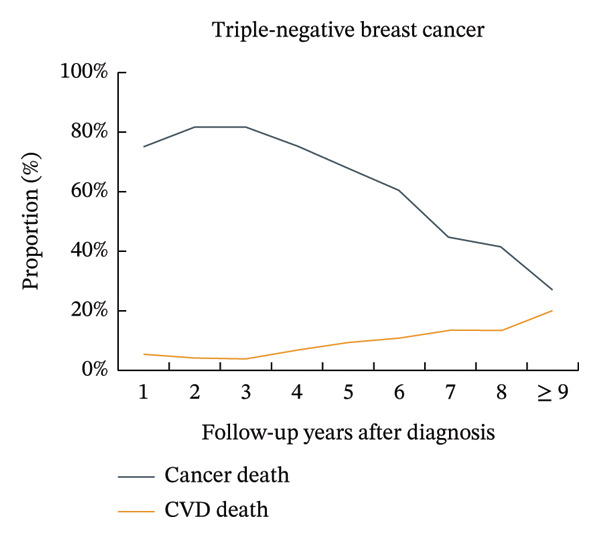
(d)

### 3.3. Cumulative Mortality

The cumulative mortalities for all causes of death among patients with different subtypes of BC are illustrated in Supporting Figure 3. For patients with luminal A BC, the highest cumulative mortality of death was caused by BC, followed by other causes of death, and CVD‐specific cumulative mortality was lower than cumulative mortality due to BC and other causes of death. Similar trends could be observed among patients with luminal B, HER‐2‐enriched, and triple‐negative BC.

Patients aged less than 50 years had the highest cumulative BC mortality among all four subtypes of BC (Supporting Figure 4). However, the cumulative CVD mortality showed an increasing trend with follow‐up time and age at diagnosis. For patients with luminal A BC aged over 74 years, the cumulative CVD mortality exceeded the cumulative BC mortality at approximately 8 years after diagnosis (Figure 2). In the same age group of patients with other subtypes of BC, the cumulative CVD mortality did not exceed the cumulative BC mortality (Supporting Figure 4).

FIGURE 2Cumulative cardiovascular disease and breast cancer–specific mortality in different subtypes of breast cancer patients aged 74 years or older. The figure illustrates the cumulative cardiovascular disease and breast cancer–specific mortality in different subtypes of breast cancer patients aged 74 years or older by using cumulative incidence functions based on the Fine–Gray hypothesis. In patients over 74 years old with breast cancer, the cardiovascular mortality risk and breast cancer mortality risk of four different subtypes of breast cancer were compared over time. The numbers of luminal A, luminal B, HER‐2‐enriched, and triple‐negative breast cancer patients were 34,313, 4366, 2022, and 5840, respectively. For patients with luminal A breast cancer aged over 74 years, the cumulative cardiovascular disease mortality exceeded the cumulative breast cancer mortality at approximately 8 years after diagnosis.

**Figure:**
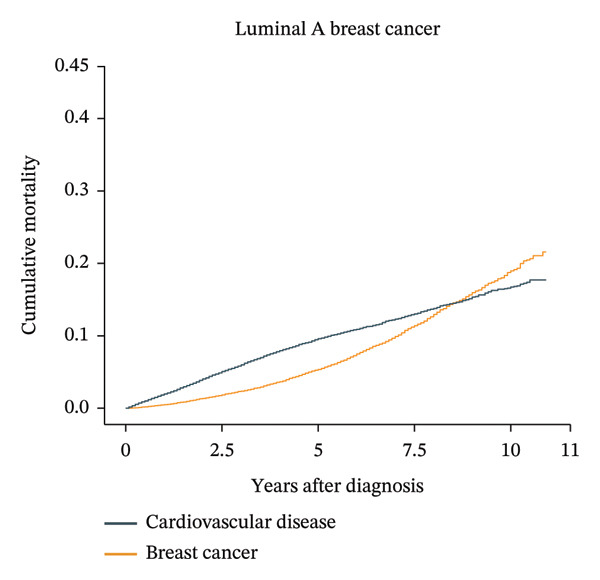
(a)

**Figure:**
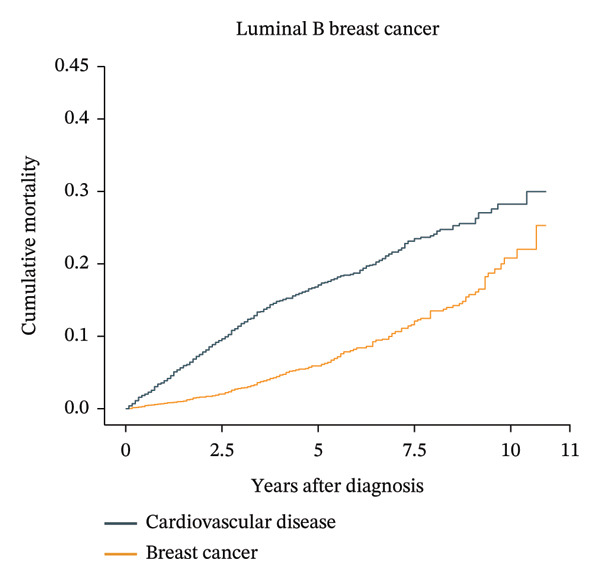
(b)

**Figure:**
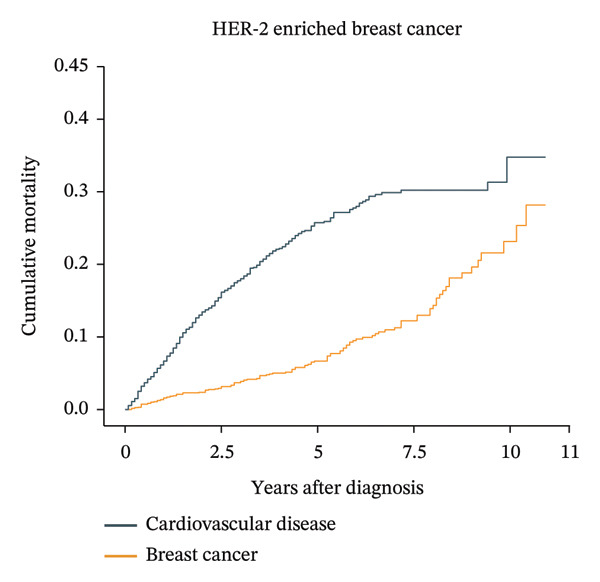
(c)

**Figure:**
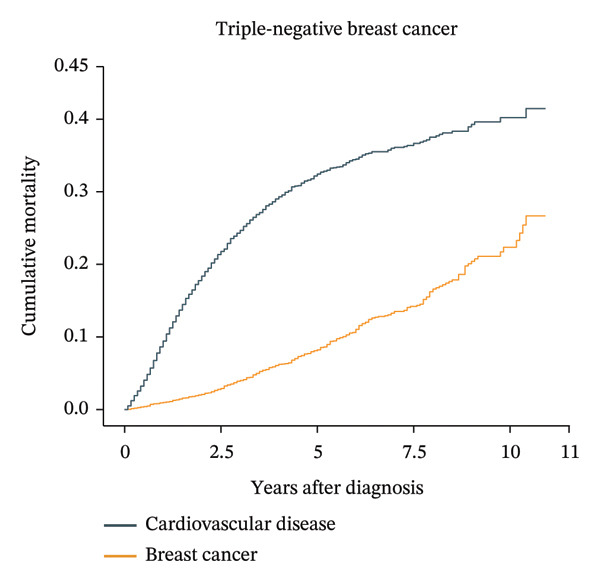
(d)

### 3.4. SMRs

When CVD mortalities among patients with different subtypes of BC were compared with those in the general female US population, the SMRs of HER‐2‐enriched and triple‐negative BC tended to increase in patients aged 55–84 years. A finer stratification of this age range, as detailed in Supporting Table 2, revealed that this elevated risk was primarily driven by patients aged 55–64 years. Within this decade, patients with triple‐negative BC (SMR 1.592 [95% CI: 1.355–1.872[) and HER‐2‐enriched BC (SMR 1.321 [95% CI: 1.124–1.552]) faced the highest excess CVD mortality compared with the general population. In patients aged older than 84 years, the SMRs tended to decrease for all subtypes of BC compared to those of the general female US population, with decreases of 39.6%, 29.5%, 33.5%, and 28.6% for patients with luminal A, luminal B, HER‐2‐enriched, and triple‐negative subtypes, respectively (Supporting Table 2). Among all BC patients, the SMR of patients aged older than 84 years tended to decrease significantly by 37% compared with that of the corresponding general female US population (Supporting Table 3).

### 3.5. CVD Mortality Risk in Different Subtypes of BC

As shown in Table 2, CVD mortality most strongly increased with age, with HRs (95% CIs) as high as 3.227 (2.781, 3.744), 9.363 (8.101, 10.822), and 30.729 (26.620, 35.473) for patients aged 50–64, 65–74, and > 74, respectively, compared with patients younger than 50 years old. Compared with that of patients with the luminal A subtype, an increased risk of CVD mortality was associated with the triple‐negative subtype (HR 1.103 [95% CI: 1.016–1.199]). Increased CVD mortality was also associated with advanced‐stage BC, with HRs of 1.241 (1.172, 1.314) and 1.224 (1.033, 1.450) for patients with regional and distant BC, respectively, compared to those with localized/in situ BC. Older age, advanced stage, and triple‐negative subtype were associated with an increased risk of BC mortality. Similar results were observed in BC patients with follow‐up times longer than 3 months (Supporting Table 4).

**TABLE 2:** Cause‐specific hazards with 95% confidence intervals for CVD‐related and breast cancer–related mortality among breast cancer patients classified by molecular subtype who received either chemotherapy or radiotherapy or both.

Variable	Total number	No. CVD deaths	CVD specific hazard ratio	No. BC deaths	BC specific hazard ratio
Age at diagnosis, years					
< 50	96,414	205	1.000 (ref.)	8561	1.000 (ref.)
50–64	173,672	1148	3.227 (2.781, 3.744)	13,582	1.037 (1.009, 1.066)
65–74	107,131	1842	9.363 (8.101, 10.822)	7036	1.082 (1.047, 1.119)
74+	46,541	2668	30.729 (26.620, 35.473)	4934	1.591 (1.525, 1.659)
Race					
White	330,970	4682	1.000 (ref.)	25,012	1.000 (ref.)
Black	47,377	860	1.651 (1.533, 1.777)	6362	1.404 (1.363, 1.446)
Other (American Indian/AK Native, Asian/Pacific Islander)	43,076	316	0.740 (0.660, 0.828)	2698	0.885 (0.851, 0.951)
Unknown	2335	5	0.303 (0.126, 0.731)	41	0.327 (0.242, 0.442)
Type of Reporting Source					
Hospital inpatient/outpatient or clinic	405,548	5546	1.000 (ref.)	32,847	1.000 (ref.)
Other/Unknown	18,210	317	1.060 (0.947, 1.187)	1266	0.872 (0.823, 0.923)
Year of diagnosis					
2010–2014	179,227	4296	1.000 (ref.)	22,035	1.000 (ref.)
2015–2020	244,531	1567	0.678 (0.637, 0.722)	12,078	0.726 (0.708, 0.745)
SEER historic stage					
Localized/In situ	256,661	3466	1.000 (ref.)	6762	1.000 (ref.)
Regional	143,040	2043	1.241 (1.172, 1.314)	15,795	4.384 (4.259, 4.513)
Distant	24,057	354	1.224 (1.033, 1.450)	11,556	28.844 (27.678, 30.059)
Molecular subtype					
Luminal A	292,655	4160	1.000 (ref.)	18,105	1.000 (ref.)
Luminal B	52,831	615	1.044 (0.959, 1.137)	3985	0.910 (0.879, 0.942)
HER‐2 enriched	22,354	291	1.069 (0.948, 1.205)	2633	1.255 (1.202, 1.310)
Triple‐negative	55,918	797	1.103 (1.016, 1.199)	9390	2.745 (2.662, 2.830)

Abbreviations: BC, breast cancer; CVD, cardiovascular disease.

When comparing CVD mortality according to HER‐2 status, being diagnosed with HER‐2‐positive BC was not significantly associated with CVD mortality (HR 1.035 [95% CI: 0.964–1.111]) compared with being diagnosed with the HER‐2‐negative subtype (Supporting Table 5). Similar results were observed in BC patients with follow‐up times longer than 3 months (Supporting Table 6).

## 4. Discussion

In this cohort of more than 0.4 million BC patients, CVD mortality was an important competing risk for all four subtypes of BC, and CVD mortality steadily increased with age. Compared with the luminal A subtype, the triple‐negative subtype was associated with an increased risk of CVD mortality.

Following advances in BC‐specific survival across four subtypes of BC survivors, potentially treatment‐induced CVD has emerged as a significant competitive risk factor for mortality, which is consistent with published studies [19, 27]. Previous studies have revealed that cardiovascular damage is associated with anticancer therapies, such as chemotherapy [28], endocrine therapy [29], and targeted therapy (such as trastuzumab) [30]. The mechanisms of cardiomyopathy associated with anthracyclines include anthracycline transport through the cell membrane of cardiomyocytes, the production of reactive oxygen species, the response to damage and repair of deoxyribonucleic acid, mitochondrial malfunction, the production of cardiotoxic anthracycline metabolites, and the disruption of sarcomeres [31]. Additionally, even if the pathophysiological mechanism underlying cardiomyocyte death for targeted therapy (such as trastuzumab) is not well understood, it has been proposed that epidermal growth factor receptor tyrosine kinase (Erb‐B2) blockade directly causes an increase in reactive oxygen species generation [30]. Additionally, cancer patients who undergo surgery might face increased cardiovascular risk. This is probably because surgery causes immediate stress on the cardiovascular system as well as prolonged inflammation after surgery [25]. Furthermore, microvascular injury induced by radiotherapy diminishes capillary density (as subacute damage), resulting in ischemia, while macrovascular injury increases age‐related atherosclerosis, leading to coronary artery disease, especially in BC patients receiving left‐sided radiotherapy [32].

An important finding of this study was that among patients with luminal A and luminal B BC, the proportions of BC mortality were still greater than those of CVD by more than 10% after 9 years of diagnosis. However, in patients with HER‐2‐enriched and triple‐negative BC, the difference between BC mortality and CVD mortality was relatively small after 9 years of diagnosis. This was most likely due to the varied treatment regimens and doses used by patients with different subtypes of BC. Additionally, compared to patients with HR‐positive BC (including luminal A and luminal B BC), patients with HR‐negative BC (including HER‐2‐enriched and triple‐negative BC) had significantly greater mortality and lower 2‐year BC‐specific survival [33]. In our study, this was reflected in a very high initial proportion of BC‐specific deaths among patients with HR‐negative disease, which underwent a rapid decline over time. Consequently, the proportion of deaths attributable to CVD progressively increased, eventually approaching that of BC after 9 years of diagnosis. This dynamic shift in mortality risks finds a plausible explanation within the competing risk framework. The population of patients with HR‐negative BC experiences substantial attrition of high‐risk individuals due to early BC‐specific mortality [34]. The surviving subpopulation that remains under follow‐up thus presents a risk profile where CVD becomes a leading cause of mortality, highlighting the imperative for dedicated cardio‐oncology survivorship strategies [9]. This led to a rapid decline in the proportion of BC mortality in patients with HR‐negative BC from the very high proportion at the beginning and eventually approached the proportion of CVD mortality after 9 years of cancer diagnosis.

Another important finding of this study was that, compared to patients with luminal A BC, patients with triple‐negative BC had a greater risk of CVD mortality. This phenomenon observed in the study is intriguing. While the exact mechanisms remain to be fully elucidated and our observational data preclude causal inferences, we can cautiously speculate on several potential pathways. First, therapeutic differences likely play a central role. Both subtypes typically necessitate aggressive, cardiotoxic chemotherapy. Triple‐negative patients rely heavily on anthracyclines and taxanes, while HER‐2‐enriched patients are exposed to the combined cardiotoxicity of anthracyclines and HER2‐targeted therapies like trastuzumab [3]. Second, the distribution of shared traditional risk factors (e.g., obesity and metabolic syndrome) may be uneven across subtypes, potentially contributing to the baseline risk [13]. Furthermore, preclinical studies suggest that mutations in genes pivotal for DNA repair and tumor suppression, such as BRCA1 and BRCA2, may also influence cardiovascular health through mechanisms like enhanced endothelial repair and reduced oxidative stress, potentially creating a biological vulnerability in carriers who develop cancer [35]. Understanding the precise underlying reasons for this disparity, whether driven primarily by treatment toxicity, shared risk factors, or tumor biology, is an important direction for future research. The results also showed that the CVD mortality risk of patients with the HER‐2‐positive subtype did not differ from that of patients with the HER2‐negative subtype, which was in line with recent studies [19, 36]. However, a recent study reported that trastuzumab‐induced CVD appeared to be reversible after early discontinuation [37]. Thus, further studies with more sophisticated designs and longer follow‐up times are needed to validate the results.

We also found that the overall CVD mortality of BC patients aged older than 84 years was lower than that of the corresponding general population. The overall CVD mortalities of BC patients aged 55–64, 65–74, and 75–84 years were comparable to those of the corresponding general population. These findings were in line with those of previous studies [8, 19]. The lower SMR for BC patients over 84 years old compared to the general population may be due to several factors. These patients have likely survived longer than average, indicating they may have less aggressive cancer or better overall health, leading to a lower mortality rate. Advances in BC treatments have improved long‐term survival, contributing to better outcomes in this age group [38]. Additionally, older patients who reach this age are often healthier and may have fewer comorbid conditions compared to the general population, which includes a broader range of health issues. Furthermore, when stratified by subtype, only the CVD mortality of HR‐negative BC patients aged 55–84 years was greater than that of the corresponding general population. This might be due to different treatment strategies across the four subtypes. The evaluation of SMRs in this study provided crucial preliminary population‐level data to aid in the development of therapeutic treatments, which might assist doctors in the management of patients at risk for increased CVD mortality.

Several limitations of this study deserve attention. First, preexisting illnesses, especially preexisting CVD, might modify the risk of CVD mortality after cancer diagnosis. Second, some risk factors shared between BC and CVD were not adjusted for in the multivariate analysis. These risk factors were not recorded in the database. Third, SMRs were not adjusted for other covariates. As a result, certain risk variables other than sex and age might have influenced these findings, and further multivariable analyses comparing patients with different subtypes of BC to non‐BC cohorts are highly desirable. Furthermore, the WONDER database provides only aggregated mortality data for the entire population, making it impossible to exclude women with a BC diagnosis. However, due to the favorable prognosis of patients with BC, the SMRs might not significantly change. Nonetheless, this study, to our knowledge, is the first to investigate the differences in intermediate‐term CVD mortality among patients with different molecular subtypes of BC. The large sample size of more than 0.4 million BC patients and relatively long follow‐up time make the results credible.

## 5. Conclusions

In conclusion, our study reveals that triple‐negative BC patients demonstrate significantly elevated CVD mortality compared to other subtypes. These findings highlight the importance of incorporating cardiovascular risk monitoring into the clinical management of these patients. The analysis further shows that HR‐negative patients aged 55–84 experience CVD mortality rates exceeding those of the general population. While detailed treatment strategies were not available in this dataset, the observed associations underscore the need for comprehensive care approaches that address both oncologic and cardiovascular health. Future research incorporating treatment details will help further elucidate these important relationships and optimize patient care strategies.

**Nomenclature**
CVD: cardiovascular disease
SEER: Surveillance, Epidemiology, and End Results
BC: breast cancer
Erb‐B2: epidermal growth factor receptor tyrosine kinases
HR: hazard ratio
SMR: standardized mortality ratio
CDC: Centers for Disease Control and Prevention

## Author Contributions

Study concept and design: Zhipeng Wang, Yacong Zhang, and Zhongyang Shen. Data acquisition, analysis, and interpretation: Zhipeng Wang, Lihong Deng, Peng Wang, and Zhongyang Shen. The first draft of the manuscript: Zhipeng Wang. The critical revision of the manuscript for important intellectual content: Lihong Deng and Zhongyang Shen. Funding: Zhongyang Shen. Study supervision: Yacong Zhang and Zhongyang Shen.

## Funding

This study was supported by the Special Funds of the National Natural Science Foundation of China (Grant number: 82241219), the National Major Scientific Research Instrument Development Project of China (Grant number: 82127808), and the Foundation for Innovative Research Groups of the National Natural Science Foundation of China (Grant number: 81921004).

## Disclosure

All authors read and approved the final manuscript.

## Ethics Statement

The authors have nothing to report.

## Consent

The authors have nothing to report.

## Conflicts of Interest

The authors declare no conflicts of interest.

## Supporting Information

Supporting Figure 1 illustrates the screening process of the study population, with a final inclusion of 423,758 patients.

Supporting Table 1 presents the baseline characteristics associated with cardiovascular mortality risk in breast cancer patients across different molecular subtypes. Fine–Gray competing risk regression models were employed to identify potential predictors of cardiovascular disease–related death in each breast cancer subtype.

Supporting Figure 2 illustrates the temporal trends in cause‐specific mortality (cardiovascular disease, breast cancer, and other causes) among breast cancer patients receiving chemotherapy, radiotherapy, or both. The figure demonstrates a progressive decline in breast cancer–related deaths over time since diagnosis, accompanied by a concurrent increase in cardiovascular disease–associated mortality.

Supporting Figure 3 illustrates the cumulative cause‐specific mortality in different subtypes of breast cancer patients by using cumulative incidence functions based on the Fine–Gray hypothesis. For patients with luminal A breast cancer, the highest cumulative mortality was caused by breast cancer, followed by other causes of death, and the cardiovascular disease–specific cumulative mortality was lower than the cumulative mortality due to breast cancer and other causes of death. Similar trends could be observed among patients with luminal B, HER‐2‐enriched, and triple‐negative breast cancer.

Supporting Figure 4 shows the cumulative cardiovascular disease–specific and breast cancer–specific mortality stratified by age at diagnosis among different BC molecular subtypes. The figure demonstrates that regardless of age group (< 50, 50–64, or 65–74 years at diagnosis), the cumulative cardiovascular disease mortality consistently surpassed breast cancer–specific mortality by the end of follow‐up.

Supporting Table 2 presents age‐specific standardized mortality ratios (SMRs) for different breast cancer molecular subtypes treated with radiotherapy or chemotherapy compared to the general US female population. In patients aged > 84 years, SMRs declined across all BC subtypes versus the general population: luminal A (−39.6%), luminal B (−38.5%), HER‐2‐enriched (−33.5%), and triple‐negative (−28.6%).

Supporting Table 3 presents age‐specific mortality ratios in breast cancer patients receiving radiotherapy/chemotherapy versus the US female population. Patients > 84 years showed a significant 37% lower mortality rate versus the general population.

Supporting Table 4 presents cause‐specific hazard ratios (95% CIs) for cardiovascular disease–related and breast cancer–related mortality among breast cancer patients receiving chemotherapy, radiotherapy, or both, stratified by molecular subtypes (analysis restricted to patients with > 3 months follow‐up). By limiting to patients with longer follow‐up (> 3 months), these results demonstrate the robustness of our findings.

Supporting Table 5 presents cause‐specific hazard ratios (95% CIs) for cardiovascular disease–related and breast cancer–related mortality in breast cancer patients stratified by HER‐2 status who underwent chemotherapy, radiotherapy, or both. The data indicate no significant association between HER‐2 status and cardiovascular disease mortality.

Supporting Table 6 presents cause‐specific hazard ratios (95% CIs) for cardiovascular disease–related and breast cancer–related mortality in HER‐2‐stratified breast cancer patients receiving chemotherapy and/or radiotherapy (analysis restricted to patients with > 3 months follow‐up). This restricted analysis confirms the robustness of our findings.

## Supporting information


**Supporting Information** Additional supporting information can be found online in the Supporting Information section.

## Data Availability

The data that support the findings of this study are available in Surveillance, Epidemiology, and End Results at https://seer.cancer.gov/, reference number 23. These data were derived from the following resources available in the public domain: SEER, https://seer.cancer.gov/.
