# Supplementary material for: A Cohort Study on Cardiovascular Disease Mortality in Breast Cancer Patients With Different Subtypes
Source: Breast J. 2026 Apr 18;2026:8076118. doi: 10.1155/tbj/8076118 (PMC13091089; doi:10.1155/tbj/8076118)
Supplement: Supplementary file 1 — Supporting Information Additional supporting information can be found online in the Supporting Information section. [file TBJ-2026-8076118-s001.docx]

**Supplementary material**


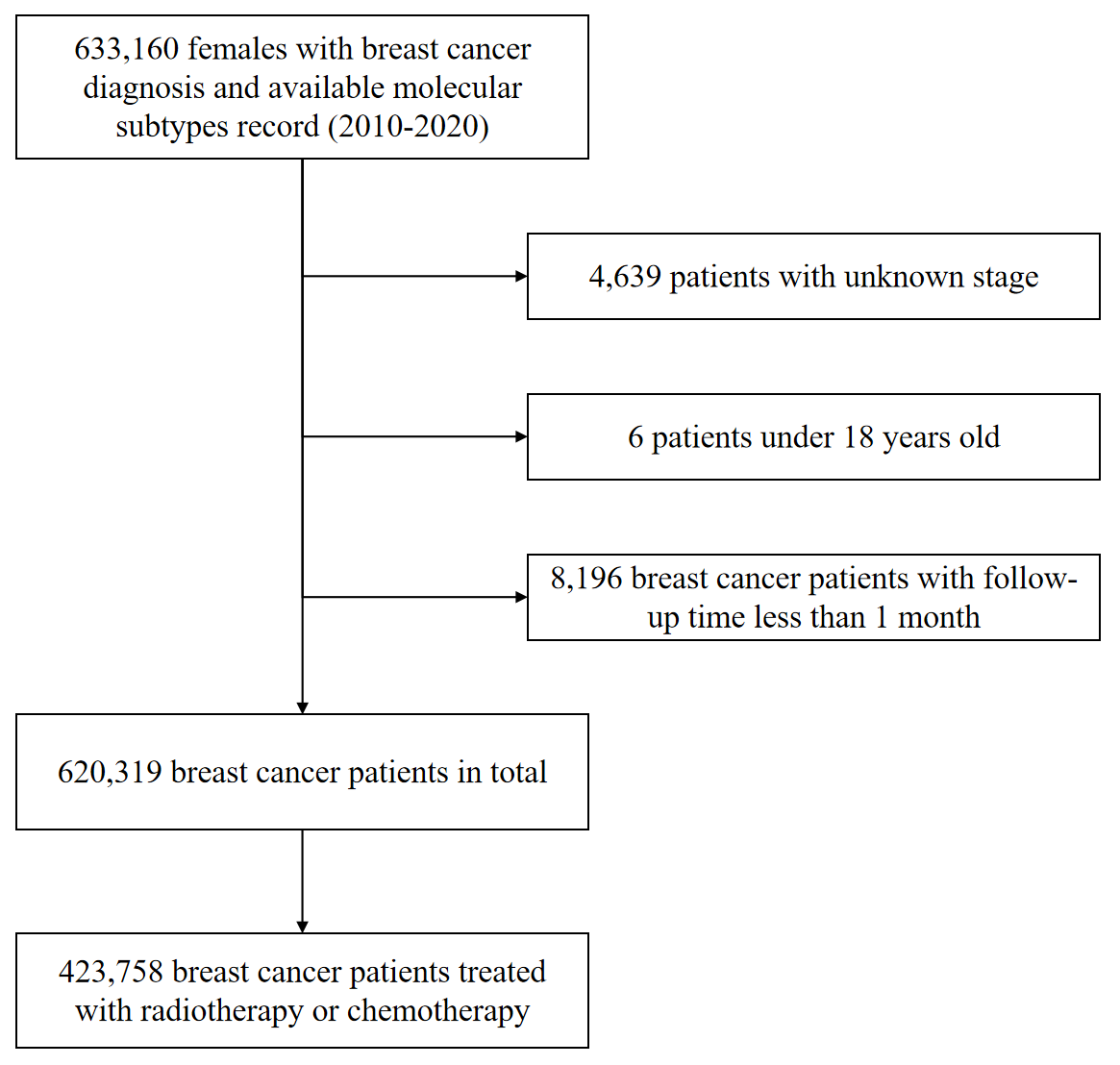


**Supplemental Figure 1. Selection of eligible patients for final analysis.**

**Supplemental Table 1. Cardiovascular mortality risk in different subtypes of breast cancer patients by baseline characteristics.**

| Variable | Luminal A BC | | |  | Luminal B BC | | |  | HER-2 enriched BC | | |  | Triple-negative BC | | |
| --- | --- | --- | --- | --- | --- | --- | --- | --- | --- | --- | --- | --- | --- | --- | --- |
|  | No. of cancer patients | CVD deaths | P value for Fine-Gray test |  | No. of cancer patients | CVD deaths | P value for Fine-Gray test |  | No. of cancer patients | CVD deaths | P value for Fine-Gray test |  | No. of cancer patients | CVD deaths | P value for Fine-Gray test |
| Age at diagnosis, years |  |  | <0.001 |  |  |  | <0.001 |  |  |  | <0.001 |  |  |  | <0.001 |
| < 50 | 58611 | 122 |  |  | 16122 | 31 |  |  | 6044 | 13 |  |  | 15637 | 39 |  |
| 50-64 | 118993 | 745 |  |  | 22030 | 138 |  |  | 10150 | 70 |  |  | 22499 | 195 |  |
| 65-74 | 80738 | 1333 |  |  | 10313 | 206 |  |  | 4138 | 82 |  |  | 11942 | 221 |  |
| 74+ | 34313 | 1960 |  |  | 4366 | 240 |  |  | 2022 | 126 |  |  | 5840 | 342 |  |
| Ethnicity |  |  | <0.001 |  |  |  | <0.001 |  |  |  | 0.003 |  |  |  | 0.013 |
| White | 235215 | 3390 |  |  | 40210 | 481 |  |  | 15917 | 220 |  |  | 39628 | 591 |  |
| Black | 26872 | 548 |  |  | 6025 | 96 |  |  | 3119 | 51 |  |  | 11361 | 165 |  |
| Other (American Indian/AK Native, Asian/Pacific Islander) | 28976 | 219 |  |  | 6314 | 38 |  |  | 3146 | 19 |  |  | 4640 | 40 |  |
| Unknown | 1592 | 3 |  |  | 282 | 0 |  |  | 172 | 1 |  |  | 289 | 1 |  |
| Type of Reporting Source |  |  | 0.223 |  |  |  | 0.023 |  |  |  | 0.607 |  |  |  | 0.009 |
| Hospital inpatient/outpatient or clinic | 279607 | 3928 |  |  | 50864 | 578 |  |  | 21444 | 280 |  |  | 53633 | 760 |  |
| Other/Unknown | 13048 | 232 |  |  | 1967 | 37 |  |  | 910 | 11 |  |  | 2285 | 37 |  |
| Year of diagnosis |  |  | 0.533 |  |  |  | 0.437 |  |  |  | 0.323 |  |  |  | 0.281 |
| 2010-2014 | 123538 | 3131 |  |  | 21802 | 424 |  |  | 9663 | 199 |  |  | 24224 | 542 |  |
| 2015-2020 | 169117 | 1029 |  |  | 31029 | 191 |  |  | 12691 | 92 |  |  | 31694 | 255 |  |
| SEER historic stage |  |  | 0.023 |  |  |  | 0.226 |  |  |  | 0.106 |  |  |  | 0.911 |
| Localized/In situ | 184280 | 2558 |  |  | 28508 | 309 |  |  | 10688 | 130 |  |  | 33185 | 469 |  |
| Regional | 94885 | 1401 |  |  | 20050 | 248 |  |  | 9186 | 119 |  |  | 18919 | 275 |  |
| Distant | 13490 | 201 |  |  | 4273 | 58 |  |  | 2480 | 42 |  |  | 3814 | 53 |  |

Note: CVD, cardiovascular disease, BC, breast cancer.


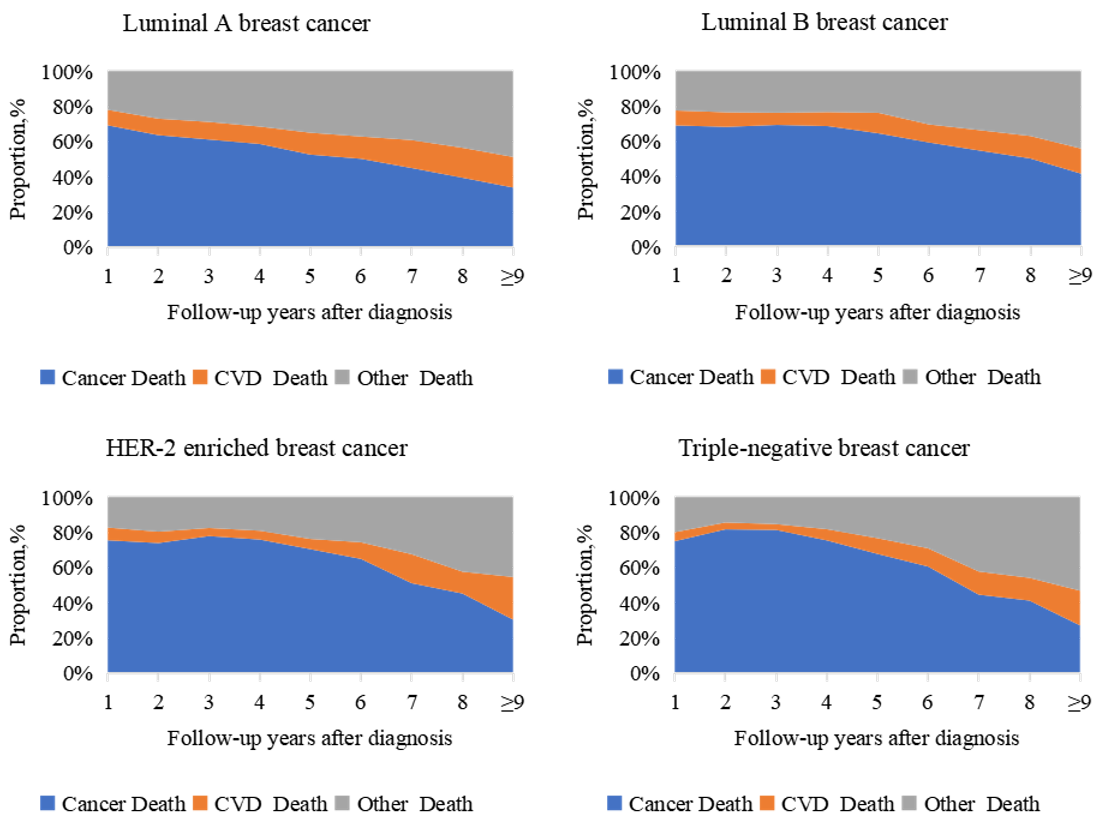


**Supplemental Figure 2. Temporal trends in the proportions of cardiovascular disease death, breast cancer death , and other causes in all-cause deaths breast cancer patients who received either chemotherapy or radiotherapy or both.**

Note: CVD, cardiovascular disease, BC, breast cancer.


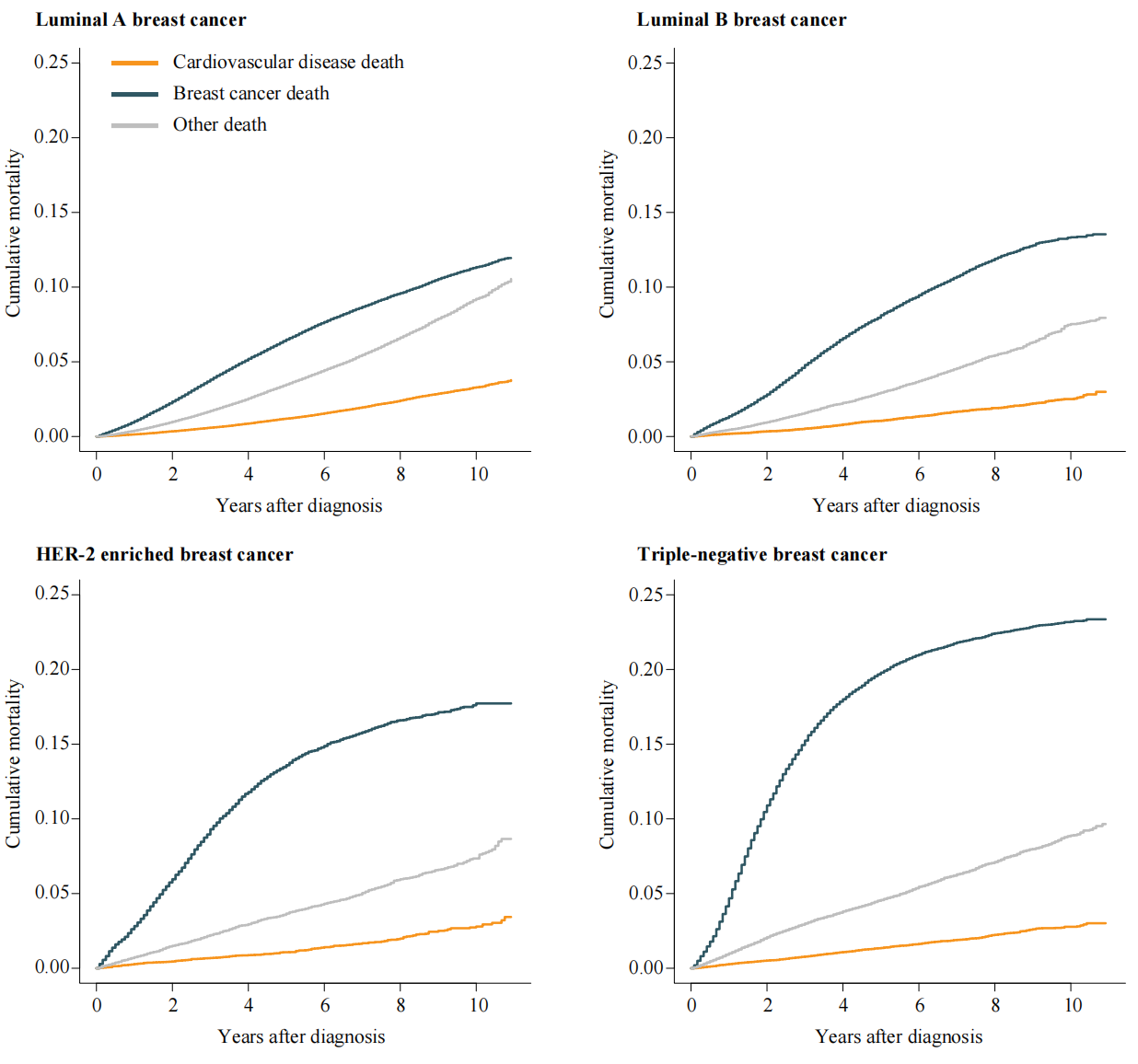


**Supplemental Figure 3. Cumulative cause-specific mortality in patients with** **different subtypes of breast cancer.** Note: CVD, cardiovascular disease.

Supplemental Figure 2 illustrates the cumulative cause-specific mortality in different subtypes of breast cancer patients by using cumulative incidence functions based on the Fine-Gray hypothesis. For patients with luminal A breast cancer, the highest cumulative mortality was caused by breast cancer, followed by other causes of death, and cardiovascular disease-specific cumulative mortality was lower than cumulative mortality due to breast cancer and other causes of death. Similar trends could be observed among patients with luminal B, HER-2-enriched and triple-negative breast cancer.


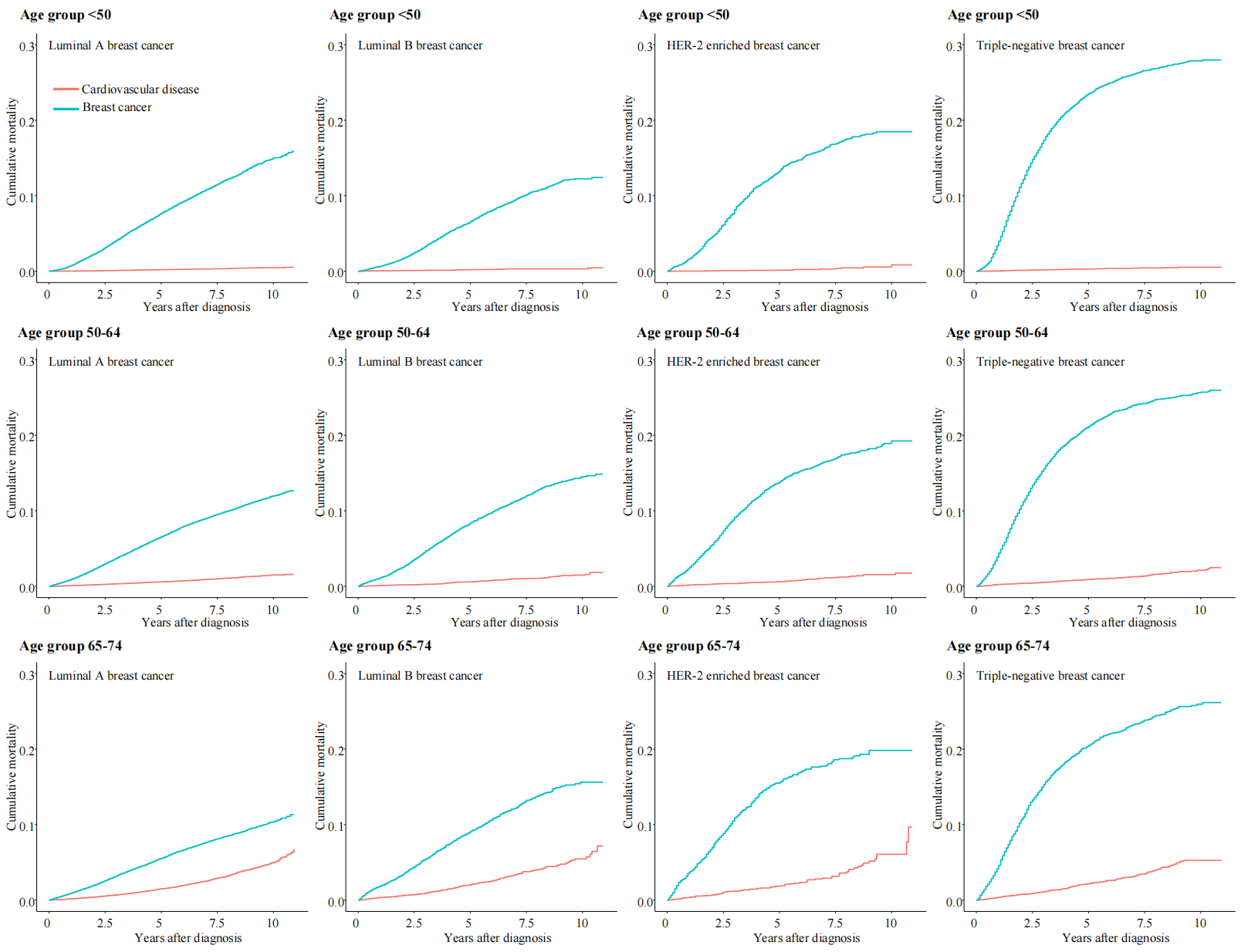


**Supplemental Figure 4. Cumulative cardiovascular disease and breast cancer-specific mortality in different subtypes of breast cancer patients by age at diagnosis**

**Supplemental Table 2 Age-specific standardized mortality ratios among different molecular subtypes of breast cancer patients who were treated with radiotherapy or chemotherapy relative to the USA female standard population.**

|  | No.CVD Death | Person year | CVD-specific mortality rate, 1/1000 person-years | Standardized mortality ratio |
| --- | --- | --- | --- | --- |
| Age group 55-64 |  |  |  |  |
| Luminal A BC | 603 | 409966 | 1.47 | 1.000 (0.851, 1.175) |
| Luminal B BC | 109 | 69720 | 1.56 | 1.063 (0.904, 1.249) |
| HER-2 enriched BC | 60 | 30884 | 1.94 | 1.321 (1.124, 1.552) |
| Triple-negative BC | 157 | 67027 | 2.34 | 1.592 (1.355, 1.872) |
| Age group 65-74 |  |  |  |  |
| Luminal A BC | 1333 | 369024 | 3.61 | 0.993 (0.896, 1.101) |
| Luminal B BC | 206 | 45723 | 4.51 | 1.239 (1.118, 1.373) |
| HER-2 enriched BC | 82 | 17687 | 4.64 | 1.275 (1.151, 1.413) |
| Triple-negative BC | 221 | 48681 | 4.54 | 1.249 (1.127, 1.384) |
| Age group 75-84 |  |  |  |  |
| Luminal A BC | 1468 | 133406 | 11.00 | 0.915 (0.865, 0.968) |
| Luminal B BC | 170 | 15452 | 11.00 | 0.915 (0.864, 0.968) |
| HER-2 enriched BC | 92 | 6671 | 13.79 | 1.147 (1.084, 1.213) |
| Triple-negative BC | 240 | 17090 | 14.04 | 1.168 (1.103, 1.235) |
| Age group 84+ |  |  |  |  |
| Luminal A BC | 492 | 15992 | 30.77 | 0.604 (0.588, 0.621) |
| Luminal B BC | 70 | 1952 | 35.86 | 0.705 (0.685, 0.724) |
| HER-2 enriched BC | 34 | 1005 | 33.83 | 0.665 (0.647, 0.683) |
| Triple-negative BC | 102 | 2806 | 36.35 | 0.714 (0.695, 0.734) |

Note: CVD, cardiovascular disease, BC, breast cancer.

**Supplemental Table 3 Age-specific standardized mortality ratios among overall breast cancer patients who were treated with radiotherapy or chemotherapy relative to the USA female standard population.**

| Variable | No.CVD Death | Person year | CVD-specific mortality rate, 1/1000 person-years | Standard mortality ratio |
| --- | --- | --- | --- | --- |
| Age group 55-64 | 929 | 577597 | 160.84 | 1.093 (0.930, 1.285) |
| Age group 65-74 | 1842 | 481114 | 382.86 | 1.053 (0.950, 1.167) |
| Age group 75-84 | 1970 | 172618 | 1141.25 | 0.949 (0.897, 1.004) |
| Age group 84+ | 698 | 21754 | 3208.61 | 0.630 (0.613, 0.648) |

Note: CVD, cardiovascular disease

**Supplemental Table 4. Cause-specific hazards with 95% confidence intervals for CVD related and breast cancer related mortality among breast cancer patients classified by molecular subtype who received either chemotherapy or radiotherapy or both. (including patients with follow-up time longer than 3 months)**

| Variable | Total number | NO. CVD deaths | CVD specific hazard ratio | NO. BC deaths | BC specific hazard ratio |
| --- | --- | --- | --- | --- | --- |
| Age at diagnosis, years |  |  |  |  |  |
| < 50 | 94626 | 201 | 1.000 (ref.) | 8434 | 1.000 (ref.) |
| 50-64 | 170149 | 1128 | 3.235 (2.784, 3.760) | 13192 | 1.026 (0.998, 1.055) |
| 65-74 | 104677 | 1815 | 9.424 (8.142, 10.906) | 6741 | 1.059 (1.024, 1.094) |
| 74+ | 45322 | 2635 | 31.135 (26.940, 35.983) | 4689 | 1.561 (1.498, 1.628) |
| Ethnicity |  |  |  |  |  |
| White | 324172 | 4613 | 1.000 (ref.) | 24249 | 1.000 (ref.) |
| Black | 46256 | 850 | 1.667 (1.547, 1.795) | 6153 | 1.405 (1.364, 1.448) |
| Other (American Indian/AK Native, Asian/Pacific Islander) | 42092 | 312 | 0.743 (0.663, 0.833) | 2615 | 0.882 (0.848, 0.919) |
| Unknown | 2254 | 4 | 0.247 (0.093, 0.661) | 39 | 0.321 (0.236, 0.437) |
| Type of Reporting Source |  |  |  |  |  |
| Hospital inpatient/outpatient or clinic | 396864 | 5467 | 1.000 (ref.) | 31825 | 1.000 (ref.) |
| Other/Unknown | 17910 | 312 | 1.055 (0.942, 1.181) | 1231 | 0.873 (0.825, 0.925) |
| Year of diagnosis |  |  |  |  |  |
| 2010-2014 | 178594 | 4264 | 1.000 (ref.) | 21579 | 1.000 (ref.) |
| 2015-2020 | 236180 | 1515 | 0.677 (0.636, 0.721) | 11477 | 0.726 (0.708, 0.745) |
| SEER historic stage |  |  |  |  |  |
| Localized/In situ | 251819 | 3448 | 1.000 (ref.) | 6705 | 1.000 (ref.) |
| Regional | 140418 | 2014 | 1.232 (1.164, 1.305) | 15694 | 4.395 (4.270, 4.524) |
| Distant | 22537 | 317 | 1.190 (1.012, 1.399) | 10657 | 27.751 (26.686, 28.859) |
| Molecular subtype |  |  |  |  |  |
| Luminal A | 286747 | 4118 | 1.000 (ref.) | 17608 | 1.000 (ref.) |
| Luminal B | 51704 | 600 | 1.036 (0.950, 1.128) | 3826 | 0.902 (0.871, 0.934) |
| HER-2 enriched | 21778 | 281 | 1.053 (0.932, 1.190) | 2509 | 1.246 (1.193, 1.302) |
| Triple-negative | 54545 | 780 | 1.101 (1.013, 1.196) | 9113 | 2.781 (2.700, 2.866) |

Note: BC, breast cancer; CVD, cardiovascular disease; age at diagnosis, ethnicity, type of reporting source, year of diagnosis, SEER historic stage and molecular subtype were adjusted in the multivariate competing risk regression models.

**Supplemental Table 5. Cause-specific hazards with 95% confidence intervals for CVD related and breast cancer related mortality among breast cancer patients classified by HER-2 status who received either chemotherapy or radiotherapy or both.**

| Variable | Total number | NO. CVD deaths | CVD specific hazard ratio | NO. BC deaths | BC specific hazard ratio |
| --- | --- | --- | --- | --- | --- |
| Age at diagnosis, years |  |  |  |  |  |
| < 50 | 96414 | 205 | 1.000 (ref.) | 8561 | 1.000 (ref.) |
| 50-64 | 173672 | 1148 | 3.217 (2.773, 3.733) | 13582 | 1.011 (0.984, 1.038) |
| 65-74 | 107131 | 1842 | 9.312 (8.058, 10.761) | 7036 | 1.022 (0.989, 1.056) |
| 74+ | 46541 | 2668 | 30.587 (26.504, 35.300) | 4934 | 1.535 (1.474, 1.598) |
| Ethnicity |  |  |  |  |  |
| White | 330970 | 4682 | 1.000 (ref.) | 25012 | 1.000 (ref.) |
| Black | 47377 | 860 | 1.670 (1.552, 1.796) | 6362 | 1.598 (1.553, 1.645) |
| Other (American Indian/AK Native, Asian/Pacific Islander) | 43076 | 316 | 0.741 (0.661, 0.830) | 2698 | 0.883 (0.849, 0.918) |
| Unknown | 2335 | 5 | 0.303 (0.126, 0.732) | 41 | 0.344 (0.255, 0.465) |
| Type of Reporting Source |  |  |  |  |  |
| Hospital inpatient/outpatient or clinic | 405548 | 5546 | 1.000 (ref.) | 32847 | 1.000 (ref.) |
| Other/Unknown | 18210 | 317 | 1.059 (0.946, 1.185) | 1266 | 0.874 (0.827, 0.924) |
| Year of diagnosis |  |  |  |  |  |
| 2010-2014 | 179227 | 4296 | 1.000 (ref.) | 22035 | 1.000 (ref.) |
| 2015-2020 | 244531 | 1567 | 0.680 (0.638, 0.724) | 12078 | 0.726 (0.708, 0.744) |
| SEER historic stage |  |  |  |  |  |
| Localized/In situ | 256661 | 3466 | 1.000 (ref.) | 6762 | 1.000 (ref.) |
| Regional | 143040 | 2043 | 1.243 (1.175, 1.316) | 15795 | 4.358 (4.234, 4.485) |
| Distant | 24057 | 354 | 1.233 (1.040, 1.461) | 11556 | 29.119 (28.057, 30.221) |
| Molecular subtype |  |  |  |  |  |
| HER-2 (-) | 348573 | 4957 | 1.000 (ref.) | 27495 | 1.000 (ref.) |
| HER-2 (+) | 75185 | 906 | 1.035 (0.964, 1.111) | 6618 | 0.793 (0.772, 0.816) |

Note: BC, breast cancer; CVD, cardiovascular disease; age at diagnosis, ethnicity, type of reporting source, year of diagnosis, SEER historic stage and molecular subtype were adjusted in the multivariate competing risk regression models.

**Supplemental Table 6. Cause-specific hazards with 95% confidence intervals for CVD related and breast cancer related mortality among breast cancer patients classified by HER-2 status who received either chemotherapy or radiotherapy or both. (only including patients with follow-up time longer than 3 months)**

| Variable | Total number | NO. CVD deaths | CVD specific hazard ratio | NO. BC deaths | BC specific hazard ratio |
| --- | --- | --- | --- | --- | --- |
| Age at diagnosis, years |  |  |  |  |  |
| < 50 | 94626 | 201 | 1.000 (ref.) | 8434 | 1.000 (ref.) |
| 50-64 | 170149 | 1128 | 3.226 (2.776, 3.749) | 13192 | 0.998 (0.972, 1.026) |
| 65-74 | 104677 | 1815 | 9.373 (8.100, 10.847) | 6741 | 0.997 (0.965, 1.031) |
| 74+ | 45322 | 2635 | 30.989 (26.820, 35.805) | 4689 | 1.501 (1.442, 1.562) |
| Ethnicity |  |  |  |  |  |
| White | 324172 | 4613 | 1.000 (ref.) | 24249 | 1.000 (ref.) |
| Black | 46256 | 850 | 1.685 (1.565, 1.814) | 6153 | 1.604 (1.558, 1.651) |
| Other (American Indian/AK Native, Asian/Pacific Islander) | 42092 | 312 | 0.744 (0.664, 0.834) | 2615 | 0.880 (0.846, 0.916) |
| Unknown | 2254 | 4 | 0.248 (0.093, 0.662) | 39 | 0.339 (0.249, 0.460) |
| Type of Reporting Source |  |  |  |  |  |
| Hospital inpatient/outpatient or clinic | 396864 | 5467 | 1.000 (ref.) | 31825 | 1.000 (ref.) |
| Other/Unknown | 17910 | 312 | 1.053 (0.940, 1.180) | 1231 | 0.875 (0.828, 0.926) |
| Year of diagnosis |  |  |  |  |  |
| 2010-2014 | 178594 | 4264 | 1.000 (ref.) | 21579 | 1.000 (ref.) |
| 2015-2020 | 236180 | 1515 | 0.678 (0.637, 0.723) | 11477 | 0.726 (0.708, 0.744) |
| SEER historic stage |  |  |  |  |  |
| Localized/In situ | 251819 | 3448 | 1.000 (ref.) | 6705 | 1.000 (ref.) |
| Regional | 140418 | 2014 | 1.235 (1.166, 1.307) | 15694 | 4.364 (0.424, 4.492) |
| Distant | 22537 | 317 | 1.196 (1.017, 1.407) | 10657 | 27.844 (26.867, 28.856) |
| Molecular subtype |  |  |  |  |  |
| HER-2 (-) | 341292 | 4898 | 1.000 (ref.) | 26721 | 1.000 (ref.) |
| HER-2 (+) | 73482 | 881 | 1.025 (0.954, 1.102) | 6335 | 0.786 (0.764, 0.808) |

Note: BC, breast cancer; CVD, cardiovascular disease; age at diagnosis, ethnicity, type of reporting source, year of diagnosis, SEER historic stage and molecular subtype were adjusted in the multivariate competing risk regression models.
